# Estimating transmission dynamics and serial interval of the first wave of COVID-19 infections under different control measures: a statistical analysis in Tunisia from February 29 to May 5, 2020

**DOI:** 10.1186/s12879-020-05577-4

**Published:** 2020-12-02

**Authors:** Khouloud Talmoudi, Mouna Safer, Hejer Letaief, Aicha Hchaichi, Chahida Harizi, Sonia Dhaouadi, Sondes Derouiche, Ilhem Bouaziz, Donia Gharbi, Nourhene Najar, Molka Osman, Ines Cherif, Rym Mlallekh, Oumaima Ben-Ayed, Yosr Ayedi, Leila Bouabid, Souha Bougatef, Nissaf Bouafif ép Ben-Alaya, Mohamed Kouni Chahed

**Affiliations:** 1 National Observatory of New and Emerging Diseases, Tunis, Tunisia; 2 Research laboratory “Epidemiology and Prevention of Cardiovascular Diseases in Tunisia”, Tunis, Tunisia; 3 Department of Epidemiology and Statistics, Abderrahman Mami Hospital, Ariana, Tunisia; 4 Department of Epidemiology and Public Health, Faculty of Medicine of Tunis, Tunis El Manar University, Tunis, Tunisia

**Keywords:** Coronavirus, Reproduction number, Serial interval, Lockdown, Statistical models, Tunisia

## Abstract

**Background:**

Describing transmission dynamics of the outbreak and impact of intervention measures are critical to planning responses to future outbreaks and providing timely information to guide policy makers decision. We estimate serial interval (SI) and temporal reproduction number (R_t_) of SARS-CoV-2 in Tunisia.

**Methods:**

We collected data of investigations and contact tracing between March 1, 2020 and May 5, 2020 as well as illness onset data during the period February 29–May 5, 2020 from National Observatory of New and Emerging Diseases of Tunisia. Maximum likelihood (ML) approach is used to estimate dynamics of R_t_.

**Results:**

Four hundred ninety-one of infector-infectee pairs were involved, with 14.46% reported pre-symptomatic transmission. SI follows Gamma distribution with mean 5.30 days [95% Confidence Interval (CI) 4.66–5.95] and standard deviation 0.26 [95% CI 0.23–0.30]. Also, we estimated large changes in R_t_ in response to the combined lockdown interventions. The R_t_ moves from 3.18 [95% Credible Interval (CrI) 2.73–3.69] to 1.77 [95% CrI 1.49–2.08] with curfew prevention measure, and under the epidemic threshold (0.89 [95% CrI 0.84–0.94]) by national lockdown measure.

**Conclusions:**

Overall, our findings highlight contribution of interventions to interrupt transmission of SARS-CoV-2 in Tunisia.

**Supplementary Information:**

**Supplementary information** accompanies this paper at 10.1186/s12879-020-05577-4.

## Introduction

Since December 2019, the epidemic of the novel severe acute respiratory syndrome coronavirus 2 (SARS-CoV-2), the causative agent of coronavirus disease 2019 (COVID-19) has been spreading. Initially present in Wuhan, China [1], it was officially declared a pandemic on March 11, 2020 by the World Health Organization [2].

In Tunisia, as of January 22, 2020, government has implemented early prevention measure, including screening in point of entry and systematic 14 days isolation of travelers returning from risk areas. The first confirmed case, among an international traveler from Italy, was reported on March 2, 2020. One week later, Tunisian government reinforce its suppression strategy with additional preventive measures. Following the reporting of 13 new cases on March 12, 2020 closure of school and university facilities was announced. The government announced further prevention measures, specifically border closure with Italy as of 14 March. On March 17, 2020, a curfew throughout the whole country, starting on March 18, 2020 was decided. Also, the closure of all sea and air borders were applied as of March 18, 2020. On March 20, a national lockdown, with a ban of transport between governorates were announced from 22 March. Finally, transition to the risk level 3 was announced on March 22, 2020.

Data are accumulating daily on transmission of COVID-19 virus in Tunisia. These data are vitally important in controlling the spread of this virus and settle the current pandemic. Determination of the serial interval (SI), the time between the symptoms onset in the primary patient (infector or index case) and symptoms onset in the patient receiving that infection from the infector (the infectee or secondary case) is fundamental in estimating the basic reproduction number (R_0_), which is the number of infectees resulting from one infector throughout his entire infectious period [3, 4].

Besides, the temporal reproduction number *R*_*t*_ is one of the key parameters in public health because it determines the extent of the epidemic, i.e. it characterizes the number of infected people caused by a contagious person during the period of his infection; it summarizes the potential transmissibility of the disease and indicates whether an epidemic is under control.

Up to now, the reproduction number (R_t_) can only been estimated retrospectively for periods from which all secondary cases had been detected. In terms of policy making and evaluation during outbreaks, obtaining estimates of the temporal tendency in the reproduction number covering as recent a time as possible would be critical [5].

R_t_ is the only reproduction number easily estimated in real time [6]. Moreover, effective control measures undertaken at time *t* are expected to result in a sudden decrease in R_t_. Hence, assessing the impact of public health interventions to mitigate the disease is easier by using estimates of R_t_. For these reasons, we focus on estimating the instantaneous reproduction number R_t_ in Tunisia.

## Material and methods

### Data

Data of SARS-CoV-2 were collected from the National Observatory of New and Emerging Diseases of Tunisia. The first dataset consists of time series of symptom onset reported from February 29, 2020, to May 5, 2020. The second dataset is obtained from contact tracing between March 1 and May 5, 2020. It was screened to clearly identified transmission events, which are a known pairs of index and secondary cases and the dates of symptom onset for both cases. Data were anonymized for this study, we only report the certain pairs of infector/infectee during the study period.

### Inference methods

A two-step procedure is used to estimate the Rt. It consists of the use of data informing the SI and daily temporal incidence onset of cases data [7]. The first step uses data on known pairs of index (infector) and secondary (infectee) cases to estimate the SI distribution; the second step estimates the time-varying reproduction number jointly from disease onset time series and from the SI distribution fitted in the first step.

#### Estimation of the serial interval distribution

Serial intervals distribution can be estimated during an ongoing outbreak using data from the list of censored lines by interval, i.e. the lower and upper limits of the date of symptom onset in index and secondary cases [8]. For each infector/infectee pair, a delay between the date of symptom onset, as claimed by the infector, and the date of symptom onset, as claimed by the infected person, is calculated [9]. In some cases, the infected person develops symptoms before the person transmitting the virus, in this case the difference between two dates will be negative.

Maximum likelihood (ML) estimates and the Akaike information criterion (AIC) are used to evaluate widely used parametric candidate models for the SARS-CoV-2 serial interval distributions namely normal, lognormal, Weibull, and gamma. Since our SI data includes a considerable number of non-positive values, we fit the four distributions both to positive values (truncated) and to shifted data, in which 12 delays are added to each observation [9]. However, caution against making assessments and projections based on the truncated data should be carefully explored and we do not believe there is cause for excluding the non-positive data.

#### Estimation of the number of temporal reproduction

At the beginning of an epidemic, when the whole population is susceptible (i.e. not immune), this number takes on a particular value denoted R_0_ and called basic reproduction number [10].

The calculation of R_0_ is based on three underlying assumptions as follows:

1. Screening strategy in Tunisia is assumed to be constant,
2. Spatial structure is neglected,
3. Incidences used are those available since February 29, 2020 and until March 18, 2020 (date of the curfew) for R_0_ and until 5 May 2020 for temporal reproduction number.

During the outbreak, when the proportion of immunized persons becomes sufficiently large to slow the transmission of the virus (by an effect similar to a reduction in the number of individuals still susceptible), we speak about the effective, or temporal, reproduction number denoted R_t._ [11]

Analyses for estimating the reproduction number were conducted using the *EpiEstim* [7, 12, 13] package on the R statistical software (version 3.6.3) [14]. This package is based on an approach that is motivated by the fact that in the situation where the epidemic under study would still be ongoing, and more particularly when it comes to evaluating the effectiveness of control measures, the total number of infections caused by the latest cases detected is not yet known. For *EpiEstim* package, the highlighted approach to temporal reproduction number leads to the instantaneous reproduction number, which is prospective: its calculation is based on the potential number of secondarily infected persons that a cohort of cases could have caused if the conditions of transmissibility had remained the same as at the time of their detection.

Let’s denote the total cases by symptom onset arising at time-step *t* by I_t_ (assuming total cases of local and imported). Following [6, 7], in which the time-dependent reproduction number, R_t_, is illustrated as the ratio of the number of new infected cases at time t, I_t_, and the total infection potential across all infected individuals at time unit t, Λ_t_. If there is a single serial interval distribution ω_s_ (s = 1,2,...), representing the probability of a secondary case arising a time period s after the primary case, each incident case that appeared at a previous time-step t-s contributes to the current infectiousness at a relative level given by ω_s_. Therefore conditional on ω_s_, Λ_t_ can be computed as follows:

$$ {\Lambda}_{\mathrm{t}}\left({\mathrm{w}}_{\mathrm{s}}\right)=\sum \limits_{\mathrm{s}=1}^{\mathrm{t}}{\mathrm{I}}_{\mathrm{t}-\mathrm{s}}{\mathrm{w}}_{\mathrm{s}} $$

Formally the *EpiEstim* package maximizes the likelihood of incidence data (seen as a Poisson count) observed over a time window of size τ ending at t. The assumption made here is that the reproduction number is constant over this time window [t-τ,t]. The estimation of the reproduction number at each time window, denoted R_t,τ_, for the time interval [t-τ,t] verifies:

$$ {R}_{t,\tau }(t)=\underset{R_t}{\mathrm{argmax}}\prod \limits_{k=t-\tau}^t\frac{{\left({R}_t{\Lambda}_k\left({w}_s\right)\right)}^{I_k}\mathit{\exp}\left(-{R}_t{\Lambda}_k\left({w}_s\right)\right)}{I_k!} $$

Thereby, an estimation of R_t_ is obtained given both the incidence and serial interval data, from which the mean and 95% intervals of R_t_ can be computed. The proposed formula may also be used for early detection of the effect of control measures to prevent the spread of the virus on the incidence of new cases.

For the next parts of the document, R_t_ is denoted R for simplicity. If R > 1, then one person infects more than one person, on average and the epidemic is growing. As the epidemic spreads, R decreases as an increasing proportion of the population becomes immune. When the threshold for group immunity is exceeded, R drops below 1, an epidemic peak is reached and the epidemic decreases. Public health control measures can also decrease R and thus reach epidemic peak before the threshold of population immunity is reached. Therefore, knowing the value of R at time t is essential to determine the status of the epidemic.

Moreover, the overall infectivity due to previously infected individuals of an outbreak at time t, denoted λ_t_, is a relative measure of the current force of infection. It is calculated as the sum the previously infected individuals I_t_, weighted by their infectivity at time t and is given by:

$$ {\lambda}_t=\sum \limits_{k=1}^{t-1}{I}_{t-k}{w}_k $$

The critical parameter for these calculations is the distribution of SI. If λ is falling, then that’s good: if not, bad.

## Results

### Distribution of serial interval

Contact tracing data, collected in the study period (between Feb 29 and May 5, 2020) included 188 unique infectors, with 117 index cases (infectors) who infected multiple people and 39 individuals that appear as both infector and infectee. Notably, 71 of the 491 (14.46%) reported cases have negative values in number of days separating symptom onset date for the infector and symptom onset date for the infected person. This indicates that the infected case developed symptoms earlier than the infector. Thus, the results suggest contamination during the asymptomatic phase or pre-symptomatic phase of the infected source may be occurring, i.e., infected persons may be infectious before their symptoms appear.

The SI estimates by fitting the four parametric distributions both to positive values (truncated) and to shifted data show that the gamma distribution provides the best fit for the truncated data (followed closely by the Weibull and lognormal). Fitted distributions can be found in the Additional file: Fig. S1 and Table S1. Also, the SI was estimated using the full dataset. Again, the gamma distribution can provide the best fit for the full dataset (shifted or truncated) and thus is the distribution we recommend for future epidemiological assessments and planning. Results from gamma distribution estimated mean SI of 5.30 days [95% CI 4.66–5.95] with a standard deviation (SD) of 0.26 [95% CI 0.23–0.30] for SARS-CoV-2 in Tunisia.

### Calculating R

**Fig. 1:**
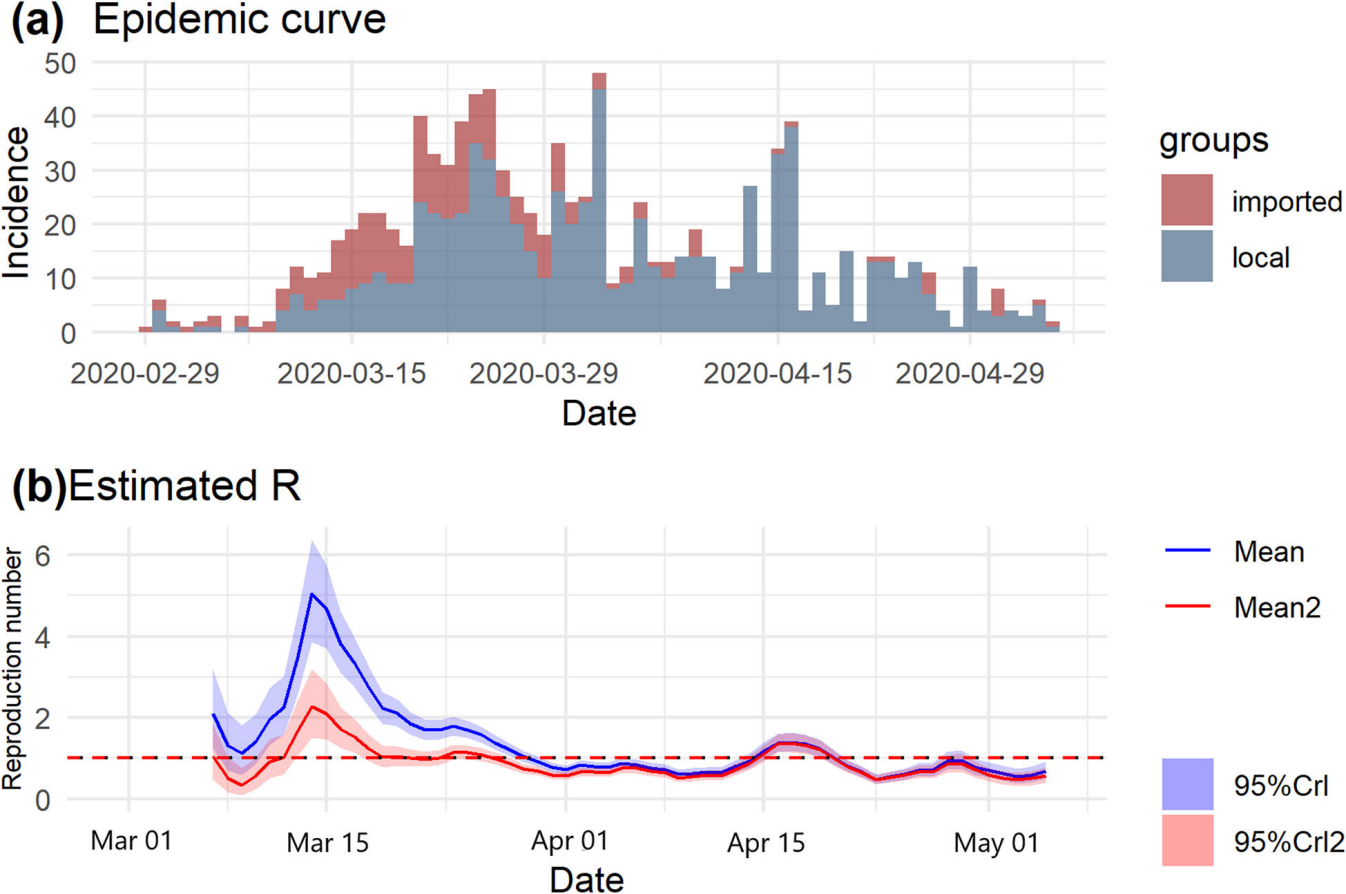
Instantaneous effect reproduction number for SARS-CoV-2 by symptom onset date in Tunisia. In the first graph is shown the daily symptom onset time series for coronavirus from February 29, 2020–May 5, 2020. The second graph shows the estimated reproduction number over sliding weekly windows (posterior mean and 95% credible interval, with estimates for a time window plotted at the end of the time window); the blue color is for all cases and the red color is for local cases; the solid lines show the posterior means and the transparent zones show the 95% credible intervals; the horizontal dashed red line indicate the threshold value R = 1

As of May 5, 2020, 1028 SARS-CoV-2 symptom onset data were reported with 246 (23.9%) being imported. In Tunisia, the epidemic started with one imported case from Italy, followed by cases with travel history and known contacts with imported cases (Fig. 1a). In March (week 1 to mid-5), the largest part of cases were local with no traveling activity (353 of 562, or 63%). For this wave of the epidemic (Fig. 1b), the R for total cases was above the epidemic threshold (> 1). For each day t of the epidemic, we estimated weekly window R ending on that day (Fig. 1b). Estimates are not shown from the very beginning of the epidemic because precise estimation is not possible in this period. R initially increased from a median value of 2.06 [95% CrI 1.36–2.97] early in the second week to 3.46 [95% CrI 2.70–4.35] at the end of the same week. R increased in the third week, reaching a peak of 5.01 [95% CrI 4.03–6.13] in the beginning of this week. Note that in this wave the estimated reproduction number for local cases (estimated as 2.25 [95% CrI 1.62–3.03] in the beginning of week 3) is, as expected, much lower than estimated when assuming that all cases were linked by local transmission (estimated as 5.01 [95% CrI 4.03–6.13]). The increased values of R from weeks 2 to 3 for both all cases and local cases suggests increasing transmissibility. This may be suggested by the existence of early “superspreaders”.

At the start of week 4, R decreased to 1.69 [95% CrI 1.49–1.90]. In April (mid-week 5 to 9), almost all cases had local transmission with few travel history (413 of 450, or 92%). R continue to decrease till the middle of week 7 with values under the epidemic threshold (< 1). Then, it increased again (mid-week 7 to mid 8) up to 1.40 [95% CrI 1.21–1.60] by 17 April 2020. Finally, R decreased again to the end of period study, up to 0.68 [95% CrI 0.52–0.87]. The weekly estimates of R can be found in the Additional file: Table S2. This could reflect the impact of control measures or could be due to the depletion of susceptibles in the Tunisian population.

Besides, to reinforce our findings and provide more information on the epidemic situation in Tunisia, the overall infectivity is globally increased over the first period study, from February 29, 2020 to April 8, 2020, then it remains stable by April 18, 2020 to peak again between April 22 to April 24, 2020. Finally, it decreases during the last period study. Illustration of the overall infectivity can be found in the Additional file: Fig. S2.

### Impact of curfew and lockdown prevention measures on R

**Fig. 2:**
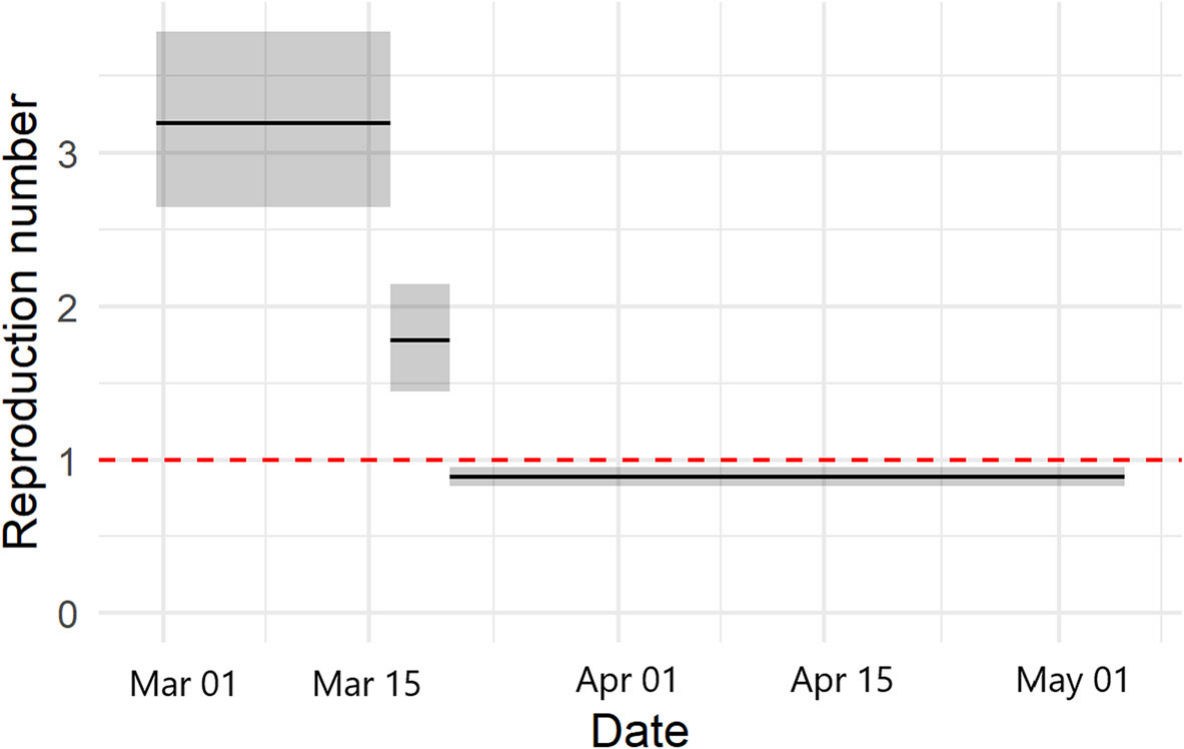
Impact of interventions on the estimates of the reproduction number R during the study period in Tunisia. The first intervention was the curfew which is applied on March 18, 2020 and the second one was the national lockdown applied on March 22, 2020

In Tunisia, the curfew was applied on March 18, 2020 and the lockdown was applied on March 22, 2020. We estimated large changes in R in response to the combined lockdown interventions. Our results suggest that the lockdown was effective in terms of reducing transmissibility (Fig. 2), as the estimated reproduction number during the lockdown was significantly lower compared to pre-intervention period. The R moves from 3.18 [95% CrI 2.73–3.69] to 1.77 [95% CrI 1.49–2.08] with curfew prevention measure, meaning that it reduces transmissibility but the risk of contagion is still alarming. By national lockdown measure, this value moves to 0.89 [95% CrI 0.84–0.94] (< 1), indicating the substantial impact of this prevention measure in reducing transmission of the epidemic.

## Discussion

We analyzed the transmission dynamics of SARS-CoV-2 infection in Tunisia in the first 3 months of the epidemics where all prevention measures were implemented, especially curfew and national lockdown.

Our results focus on the use of the likelihood-based method to estimate initial R_t_ and SI. Utilizing temporal symptom onset data and contact tracing, we provide estimates of the transmissibility parameters of SARS-CoV-2 during the first wave experienced in Tunisia. These data are incorporated to provide robust estimates of transmission parameters.

Our estimates of the SI for SARS-CoV-2 in Tunisia better resemble a gamma distribution with an estimated mean of 5.30 days [95% CI 4.66–5.95]. These estimates of the mean SI are higher than the published estimates of 4.6 days [95% CI: 3.5, 5.9] among 18 certain pairs calculated at early stage of the COVID-19 epidemic [4], indicating that SARS-CoV-2 infection in Tunisia leads to slow cycles of transmission from one generation of cases to the next. Recent estimates for the mean serial interval of COVID-19 range from 3.9 days [95% CI 2.7–73] [15] and 4.0 days [95% CI 3.1–4.9] [4] to 7.5 days [95% CI 5.3–19] [3] based on data from 21, 28 and 6 pairs, respectively. Also, the mean SI in Tunisia is considerably lower than reported mean serial intervals of 8.4 days for SARS [16] and 12.6 days [17] - 14.6 days [18] for MERS. This indicates that calculations made using the SARS SI may introduce bias.

At least, two sources of bias can be potentially considered in our estimates, which are likely to cause underestimation of SARS-CoV-2 serial intervals. First, the distribution of SI varies during an epidemic, with the time separating successive cases close to the epidemic peak [9]. To provide insight, a susceptible person would probably become infected more quickly if he is surrounded by more than one infected person. Since our estimates are based on transmission cases reported during the early stages of outbreaks, such compression are not explicitly accounted and we interpret the estimates as basic serial intervals at the beginning of an epidemic. However, our estimates may reflect effective serial intervals when certain of the reported infections occurred in the amidst of growing clusters, that would be expected during a period of epidemic growth. Second, the dates of symptom onset of each infector was likely based on individual remembrance of past activities. If accuracy of the recall is impeded by time, it is highly possible that recent encounters (short serial intervals) are attributable to infected cases rather than over past encounters (longer serial intervals). This information is self-reported from infected cases. Therefore, an information or reporting bias may occur. In contrast, the reported serial intervals may be biased upwards by travel-related delays in transmission from primary cases that were infected in another countries before returning in Tunisia. In that case, if their infectious period began while still traveling, then it is very unlikely to observe early transmission events with shorter serial intervals. Given the diversity in type and reliability sources of bias when estimating the SI, particular cautions to our findings should be granted. Our results provide working hypotheses regarding the infectivity of coronavirus in Tunisia, which will need to be validated as new data become available. In our study, we used the symptom onset dates for both infector and infected confirmed cases. However, the last exposure dates to infected person(s) for pairs of infector/infectee were not considered in this study due to messing data. These dates are useful to settle if infection is occurring during the asymptomatic phase of the infection. However, negative values in delays separating symptom onset date for the infector and symptom onset date for the infected person strongly suggest infection occurring during the asymptomatic phase of SARS-CoV-2 in Tunisia. Although contamination from asymptomatic persons has not been proven before, recent studies have highlighted the existence of such transmission [19, 20]. However, contamination from asymptomatic persons lower in comparison with symptomatic cases [21]. Here, the potential impacts for the control of SARS CoV-2 are mixed. While our lower estimates for R suggest easier lockdown, the asymptomatic transmission events remains a concern. Future works are developed to provide more information of the contamination of SARS-CoV-2 during the asymptomatic phase of the infected sources in Tunisia.

In Tunisia massive prevention strategies have been applied at early stage of the SARS-CoV-2 epidemic. These strategies include implementing travel restrictions, isolation of infected individuals, active contact tracing, make quarantining compulsory, and enforcing lockdown [22]. In Tunisia, where all interventions were implemented in short time period and some of them were separated by a short time interval, these individual effects are by definition unidentifiable. Despite this, while individual impacts cannot be determined, their estimated joint impact is strongly empirically justified [23]. Our results suggest that the lockdown was effective in mitigating transmissibility of the disease, as the estimated reproduction number during the lockdown was significantly lower compared to initial-intervention period. We showed that after less than 5 weeks from the beginning of the epidemic, R dropped below the epidemic threshold. This corresponds to a decline of new cases, which is the result of herd immunity altogether with public health control measures. However, despite the dependence of infectious diseases on climate factors [24], there is no evidence so far supporting the impact of warmer climate factors in reducing the transmissibility of the disease [25].

Likewise, we estimated that there was an early decrease in *R* for the outbreak of coronavirus in Tunisia. But just estimating R does not allow us to determine whether this reflects a true reduction in transmissibility, possibly due to the national lockdown or the depletion of susceptibles. Furthermore, with R values dropping significantly, the acquisition rate of herd immunity will slow down rapidly. This indicates the ability of the virus to spread rapidly should interventions be lifted. Interestingly, just after the first peak of incidence, R decreased under 1 for almost 2 weeks, raised above 1 for 5 days, and decreased again under 1, indicating that the epidemic was not yet over; and indeed, a second peak was still to come.

Some limitations in this study can be highlighted. First, we presume all infected cases are known and consistently reported over the study. Violation of this assumption may lead to biased estimates of the SI and the reproduction number [26]. Similarly, the variation in case tracking over time may bias our estimates of the time variation of R_t_. In general, higher reporting rates can be expected in the early phase of the epidemic, with reporting fatigue becoming a factor in late phase. Second, our estimates do not incorporate regional heterogeneity probably existing in transmission patterns, or to assess its impact on overall measures of reproduction number.

Despite these limitations, the post-pandemic estimates reported in this study add to general acquisition and knowledge of coronavirus transmissibility parameters, which were previously dominated by estimates from other countries, or occasionally from other epidemics, and are often based on preliminary data. It remains important that the revision of the parameters, based on exhaustive data from different geographical scales, be integrated into the planning of mitigation strategies for future pandemics. Nevertheless, the methods used in this study would be adaptable to generate real-time estimates for future epidemics. As we continue to build epidemiological capacity in our country, urgent improvements need to be implemented such as, digitizing contact tracing, the need for rapid assessments of transmissibility of novel pathogens, in addition to disease severity, to better inform public health interventions.

## Supplementary Information

**Additional file 1: Figure S1.** Maximum likelihood distributions fit to transformed COVID-19 serial intervals (491 reported transmission events in Tunisia between March 1, 2020 and May 5, 2020). To evaluate several positive-valued distributions (lognormal, gamma and Weibull), we took two approaches to addressing the negative-valued data. First, we left truncated the data (i.e., removed all non-positive values) for (A) all infection events. Second, we shifted the data by adding 12 days to each reported serial interval for (B) all infection events. **Table S1.** Model comparison for COVID-19 serial intervals based on all 491 reported transmission events in Tunisia between March 1, 2020 and May 5, 2020. **Table S2.** Weekly window reported estimates of the reproduction number (R) during the study period in Tunisia. **Figure S2.** Overall infectivity between February 29, 2020 and May 5, 2020.

## Data Availability

The datasets generated and/or analyzed in this study are not publicly available for respect for patient privacy, but are available from the corresponding author upon reasonable request.

## Abbreviations

AIC: Akaike information criterion
CI: Confidence Interval
CrI: Credible interval
COVID-19: Causative agent of coronavirus disease 2019
ML: Maximum likelihood
R_0_: Basic reproduction number
R_t_: Temporal reproduction number
SARS-CoV-2: Severe acute respiratory syndrome coronavirus 2
SD: Standard deviation
SI: Serial interval
