# Supplementary material for: Estimating transmission dynamics and serial interval of the first wave of COVID-19 infections under different control measures: a statistical analysis in Tunisia from February 29 to May 5, 2020
Source: BMC Infect Dis. 2020 Dec 2;20:914. doi: 10.1186/s12879-020-05577-4 (PMC7708891; doi:10.1186/s12879-020-05577-4)
Supplement: Supplementary file 1 — Additional file 1: Figure S1. Maximum likelihood distributions fit to transformed COVID-19 serial intervals (491 reported transmission events in Tunisia between March 1, 2020 and May 5, 2020). To evaluate several positive-valued distributions (lognormal, gamma and Weibull), we took two approaches to addressing the negative-valued data. First, we left truncated the data (i.e., removed all non-positive values) for (A) all infection events. Second, we shifted the data by adding 12 days to each reported serial interval for (B) all infection events. Table S1. Model comparison for COVID-19 serial intervals based on all 491 reported transmission events in Tunisia between March 1, 2020 and May 5, 2020. Table S2. Weekly window reported estimates of the reproduction number (R) during the study period in Tunisia. Figure S2. Overall infectivity between February 29, 2020 and May 5, 2020. [file 12879_2020_5577_MOESM1_ESM.docx]

**Supplementary material**


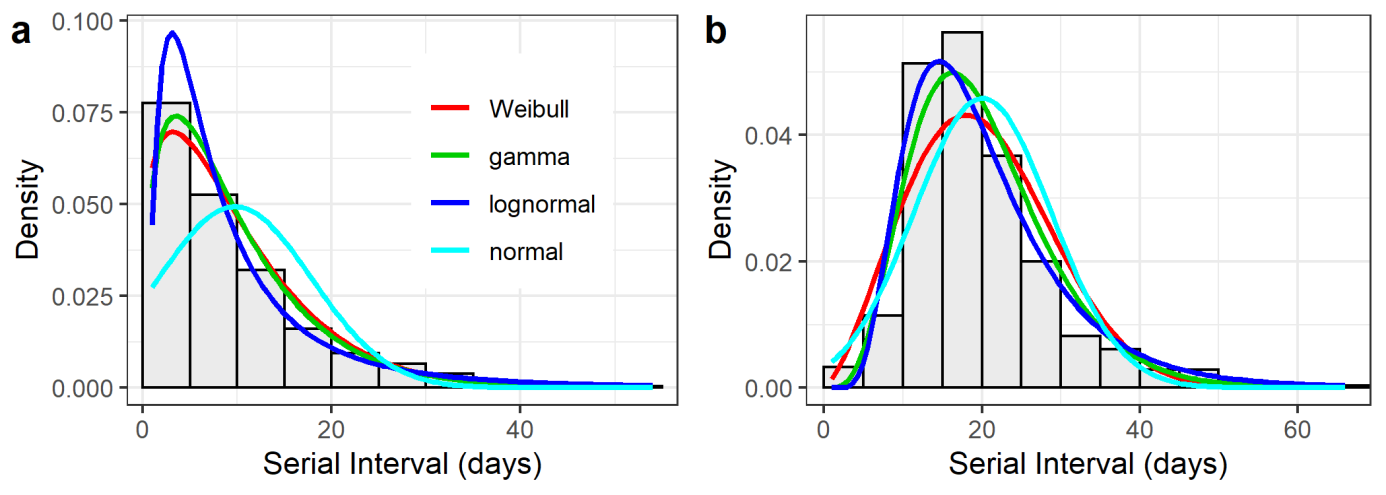


Figure S1. Maximum likelihood distributions fit to transformed COVID-19 serial intervals (491 reported transmission events in Tunisia between March 1, 2020 and May 5, 2020). To evaluate several positive-valued distributions (lognormal, gamma and Weibull), we took two approaches to addressing the negative-valued data. First, we left truncated the data (i.e., removed all non-positive values) for (A) all infection events. Second, we shifted the data by adding 12 days to each reported serial interval for (B) all infection events.

Table S1. Model comparison for COVID-19 serial intervals based on all 491 reported transmission events in Tunisia between March 1, 2020 and May 5, 2020.

| Data | Distribution | Mean/Shape [95% CI] | SD/Scale  [95% CI] | AIC |
| --- | --- | --- | --- | --- |
| Original data | Normal(Mean, SD) | 8.06 [7.29-8.83] | 8.70 [8.16-9.25] | 3522.02 |
| Truncated (>0) | Normal(Mean, SD) | 9.77 [9.00-10.54] | 8.08 [7.54-8.63] | 2972.49 |
|  | Lognormal (Shape, Scale) | 1.93 [1.84-2.01] | 0.90 [0.84-0.96] | 2744.00 |
|  | Gamma (Shape, Scale) | 1.57 [1.38-1.76] | 0.16 [0.14-0.18] | 2732.67 |
|  | Weibull (Shape, Scale) | 1.27 [1.18-1.37] | 10.58 [9.74-11.41] | 2740.18 |
| Shifted (+12d) | Normal(Mean, SD) | 21.06 [19.29-20.83] | 8.70 [8.16-9.25] | 3522.02 |
|  | Lognormal (Shape, Scale) | 2.90 [2.86-2.94] | 0.47 [0.44-0.50] | 3514.06 |
|  | Gamma (Shape, Scale) | 5.30 [4.66-5.95] | 0.26 [0.23-0.30] | 3458.36 |
|  | Weibull (Shape, Scale) | 2.39 [2.24-2.55] | 22.61 [21.72-23.49] | 3487.46 |

Table S2. Weekly window reported estimates of the reproduction number ($\boldsymbol{R}$) during the study period in Tunisia.

| Weekly window | R (All cases) | R (local) | Weekly window | R | R (local) | Weekly window | R | R (local) |  |
| --- | --- | --- | --- | --- | --- | --- | --- | --- | --- |
| 29/02-07/03 | 2.06 [1.36-2.97] | 1.01 [0.55-1.68] | 20/03-27/03 | 1.38 [1.24-1.53] | 0.99 [0.87-1.12] | 09/04-16/04 | 1.37 [1.19-1.57] | 1.34 [1.17-1.54] |  |
| 01/03-08/03 | 1.28 [0.78-1.96] | 0.47 [0.20-0.92] | 21/03-28/03 | 1.21 [1.09-1.35] | 0.87 [0.77-0.99] | 10/04-17/04 | 1.40 [1.21-1.60] | 1.37 [1.18-1.57] |  |
| 02/03-09/03 | 1.09 [0.66-1.67] | 0.32 [0.12-0.67] | 22/03-29/03 | 1.04 [0.93-1.16] | 0.74 [0.65-0.84] | 11/04-18/04 | 1.35 [1.17-1.55] | 1.32 [1.14-1.52] |  |
| 03/03-10/03 | 1.38 [0.93-1.96] | 0.54 [0.28-0.92] | 23/03-30/03 | 0.91 [0.82-1.02] | 0.68 [0.60-0.77] | 12/04-19/04 | 1.24 [1.07-1.43] | 1.22 [1.05-1.41] |  |
| 04/03-11/03 | 1.95 [1.42-2.60] | 0.90 [0.56-1.36] | 24/03-31/03 | 0.78 [0.69-0.87] | 0.58 [0.50-0.66] | 13/04-20/04 | 1.00 [0.86-1.16] | 0.99 [0.84-1.14] |  |
| 05/03-12/03 | 2.22 [1.67-2.88] | 1.01 [0.66-1.47] | 25/03-01/04 | 0.71 [0.63-0.80] | 0.56 [0.48-0.64] | 14/04-21/04 | 0.80 [0.68-0.93] | 0.78 [0.67-0.91] |  |
| 06/03-13/03 | 3.46 [2.70-4.35] | 1.65 [1.14-2.28] | 26/03-02/04 | 0.83 [0.73-0.93] | 0.67 [0.59-0.76] | 15/04-22/04 | 0.66 [0.55-0.77] | 0.64 [0.54-0.76] |  |
| 07/03-14/03 | 5.01 [4.03-6.13] | 2.25 [1.62-3.03] | 27/03-03/04 | 0.80 [0.71-0.90] | 0.65 [0.57-0.75] | 16/04-23/04 | 0.48 [0.39-0.58] | 0.46 [0.37-0.57] |  |
| 08/03-15/03 | 4.65 [3.84-5.56] | 2.08 [1.56-2.71] | 28/03-04/04 | 0.78 [0.69-0.88] | 0.65 [0.56-0.85] | 17/04-24/04 | 0.54 [0.44-0.65] | 0.52 [0.43-0.63] |  |
| 09/03-16/03 | 3.81 [3.22-4.47] | 1.71 [1.32-2.16] | 29/03-05/04 | 0.87 [0.76-0.98] | 0.75 [0.65-0.85] | 18/04-25/04 | 0.60 [0.49-0.72] | 0.58 [0.48-0.70] |  |
| 10/03-17/03 | 3.33 [2.84-3.87] | 1.51 [1.19-1.89] | 30/03-06/04 | 0.84 [0.73-0.95] | 0.75 [0.65-0.86] | 19/04-26/04 | 0.71 [0.58-0.84] | 0.65 [0.54-0.79] |  |
| 11/03-18/03 | 2.75 [2.35-3.18] | 1.22 [0.97-1.52] | 31/03-07/04 | 0.76 [0.66-0.86] | 0.68 [0.58-0.78] | 20/04-27/04 | 0.71 [0.58-0.87] | 0.65 [0.53-0.80] |  |
| 12/03-19/03 | 2.22 [1.91-2.56] | 1.03 [0.82-1.26] | 01/04-08/04 | 0.72 [0.62-0.83] | 0.64 [0.55-0.74] | 21/04-28/04 | 0.92 [0.75-1.12] | 0.84 [0.68-1.03] |  |
| 13/03-20/03 | 2.11 [1.84-2.40] | 1.04 [0.86-1.25] | 02/04-09/04 | 0.60 [0.51-0.70] | 0.51 [0.42-0.60] | 22/04-29/04 | 0.95 [0.77-1.15] | 0.87 [0.70-1.07] |  |
| 14/03-21/03 | 1.84 [1.62-2.08] | 1.00 [0.83-1.18] | 03/04-10/04 | 0.63 [0.53-0.73] | 0.54 [0.45-0.64] | 23/04-30/04 | 0.77 [0.61-0.95] | 0.71 [0.56-0.89] |  |
| 15/03-22/03 | 1.69 [1.49-1.90] | 0.97 [0.82-1.13] | 04/04-11/04 | 0.65 [0.55-0.76] | 0.58 [0.49-0.68] | 24/04-01/05 | 0.70 [0.55-0.86] | 0.58 [0.45-0.73] |  |
| 16/03-23/03 | 1.70 [1.51-1.90] | 1.00 [0.86-1.16] | 05/04-12/04 | 0.64 [0.53-0.75] | 0.57 [0.47-0.68] | 25/04-02/05 | 0.62 [0.48-0.79] | 0.50 [0.37-0.65] |  |
| 17/03-24/03 | 1.79 [1.60-1.99] | 1.14 [0.99-1.28] | 06/04-13/04 | 0.79 [0.67-0.92] | 0.72 [0.61-0.85] | 26/04-03/05 | 0.54 [0.41-0.70] | 0.47 [0.35-0.62] |  |
| 18/03-25/03 | 1.70 [1.53-1.88] | 1.13 [0.99-1.28] | 07/04-14/04 | 0.93 [0.79-1.09] | 0.88 [0.74-1.03] | 27/04-04/05 | 0.59 [0.45-0.76] | 0.50 [0.37-0.65] |  |
| 19/03-26/03 | 1.58 [1.42-1.74] | 1.09 [0.96-1.23] | 08/04-15/04 | 1.17 [1.01-1.35] | 1.10 [0.94-1.28] | 28/04-05/05 | 0.68 [0.52-0.87] | 0.56 [0.41-0.74] |  |


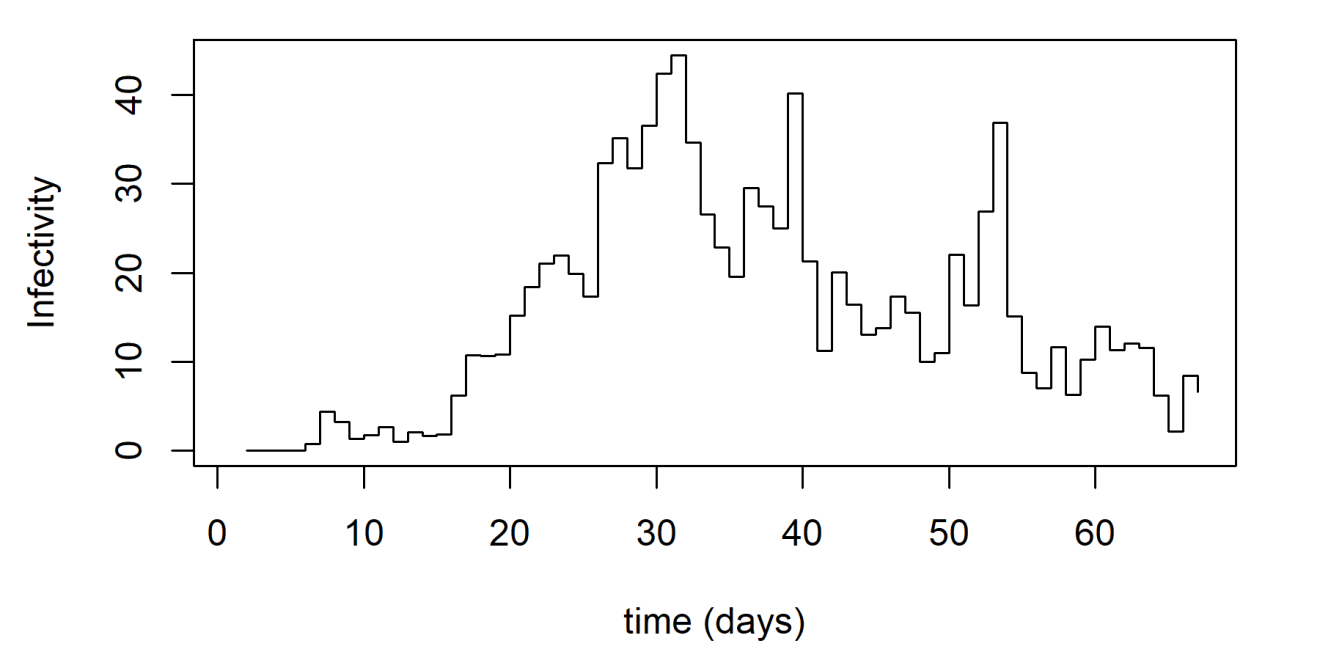


Figure S2. Overall infectivity between February 29, 2020 and May 5, 2020.
